# Temporal-Spatial Dynamics in Orthoptera in Relation to Nutrient Availability and Plant Species Richness

**DOI:** 10.1371/journal.pone.0071736

**Published:** 2013-08-12

**Authors:** Rob J. J. Hendriks, Luisa G. Carvalheiro, Roy M. J. C. Kleukers, Jacobus C. Biesmeijer

**Affiliations:** 1 Department of Ecology, Radboud University, Nijmegen, The Netherlands; 2 Institute of Integrative and Comparative Biology, University of Leeds, Leeds, United Kingdom; 3 European Invertebrate Survey-Netherlands, Leiden, The Netherlands; 4 Naturalis Biodiversity Center, Leiden, The Netherlands; University of Sussex, United Kingdom

## Abstract

Nutrient availability in ecosystems has increased dramatically over the last century. Excess reactive nitrogen deposition is known to negatively impact plant communities, e.g. by changing species composition, biomass and vegetation structure. In contrast, little is known on how such impacts propagate to higher trophic levels. To evaluate how nitrogen deposition affects plants and herbivore communities through time, we used extensive databases of spatially explicit historical records of Dutch plant species and Orthoptera (grasshoppers and crickets), a group of animals that are particularly susceptible to changes in the C:N ratio of their resources. We use robust methods that deal with the unstandardized nature of historical databases to test whether nitrogen deposition levels and plant richness changes influence the patterns of richness change of Orthoptera, taking into account Orthoptera species functional traits. Our findings show that effects indeed also propagate to higher trophic levels. Differences in functional traits affected the temporal-spatial dynamics of assemblages of Orthoptera. While nitrogen deposition affected plant diversity, contrary to our expectations, we could not find a strong significant effect of food related traits. However we found that species with low habitat specificity, limited dispersal capacity and egg deposition in the soil were more negativly affected by nitrogen deposition levels. Despite the lack of significant effect of plant richness or food related traits on Orthoptera, the negative effects of nitrogen detected within certain trait groups (e.g. groups with limited disperse ability) could be related to subtle changes in plant abundance and plant quality. Our results, however, suggest that the changes in soil conditions (where many Orthoptera species lay their eggs) or other habitat changes driven by nitrogen have a stronger influence than food related traits. To fully evaluate the negative effects of nitrogen deposition on higher trophic levels it is essential to take into account species life-history traits.

## Introduction

With the expansion of agriculture and increased use of fossil fuels, reactive nitrogen availability in ecosystems has increased dramatically over the last century [1]. In the Netherlands nitrogen availability substantially increased between 1950 and 1990, and after the implementation of environmental policy measures it started to decrease, but not down to the 1950 levels [2]. Excess reactive nitrogen causes direct and indirect damage to the functioning of ecosystems [1]. Nutrient enrichment of ecosystems may cause the decline or increase of plant species through direct toxic effects, changes in their abilities to compete for light and changes in their susceptibility to secondary stress and disturbance [3]. Such changes become visible in the vegetation in two ways: changes of species composition and changes in vegetation structure, biomass and density.

While impacts of nitrogen on plant communities have received considerable attention e.g. [4]–[5], little information is available on how such changes propagate through trophic levels. Higher trophic levels, such as herbivores and predators, may be impacted via the loss of plant species, via changes in vegetation structure or, in the case of herbivores via changes in food quality. Elevated nitrogen deposition can impact food quality by changing the internal C:N balance of plants which in turn is known to affect both the nutritional value to herbivores as well as the level of plant defence.

Here we studied the influence of excess nutrient availability on plants and Orthoptera species (grasshoppers and crickets). We used Orthoptera as a test herbivore group, as they are particularly susceptible to changes in the C:N ratio of their resources [6]–[7]. Also this group allows for comparison of herbivorous, omnivorous and carnivorous species. We studied the relationships between Orthoptera species richness, plant species richness and nitrogen deposition levels using data from the Netherlands. As the majority of Orthoptera species in the Netherlands are herbivores [8], and plant resources can be negatively affected by high levels of nitrogen e.g. [9]–[11] we expected overall changes in Orthoptera species richness to parallel changes in nitrogen deposition and changes in plant species richness both in time and space (hypothesis 1).

In addition, species responses may depend on their traits e.g. [12]. As nitrogen deposition can affect the herbivore-defence mechanisms and the nutritional value of plants [13], we expected that herbivores would be impacted more by nitrogen overload than carnivores and omnivores (hypothesis 2).

Specialist Orthoptera feed mostly on grasses. As grasses are generally impacted less negatively by increased nitrogen deposition than herbs, or even beneficiate from increases in soil nitrogen availability [14]–[17], we expected grass specialists to benefit more from nitrogen availability than generalists (hypothesis 3).

As habitat specialist species are more susceptible to habitat loss and species with great dispersal ability are more able to adjust their spatial range, we expected Orthoptera with a wide habitat range and limited dispersal ability to show a stronger relationship with changes in nitrogen deposition than habitat specialists (hypothesis 4).

Finally, since nitrogen deposition levels relate to changes in the structure and density of the vegetation and thus to the microclimate, we expected the group of Orthoptera laying their eggs in the soil to be more impacted by changes in nitrogen deposition than those who lay their eggs in plants (hypothesis 5). This because the microclimate (ground temperature) is related to the hatching success of the eggs [18]. In contrast to the above, we did not expect any specific relationship between development rate of the Orthoptera species and nitrogen deposition level.

While nitrogen deposition is known to have strong effects on plant communities, little was known on how such impacts propagate to higher trophic levels.

With this study we provide evidence of impacts of nitrogen deposition on higher trophic levels and also explore the possible mechanisms by comparing responses of groups with contrasting traits.

## Methods

No standardized recording schemes exist for the Orthoptera of the Netherlands. However, the existing large amount of haphazardly collected historical observations provides an opportunity to evaluate changes in this group e.g. [12], [19], [20]. The change in species richness is considered to represent the shifts in ecological success of a given group of species. Shifts in species richness are analysed per grid-cell by comparing the value of richness, estimated based on species accumulation curves, in different periods.

### Data Availability and Choice of Periods for Comparison

The Orthoptera database of the European Invertebrate Survey – The Netherlands and other databases included in the National Database of Flora and Fauna were used. The total number of records used was 297535.

Plant species data were also taken from the National Database on Flora and Fauna of the Netherlands. We only used the plant data in the NDFF originating from vegetation relevees [21].

As comparing unequal time periods may bias the results [19], here we compare periods of equal length (15 years). Based on the temporal distribution of nitrogen deposition in the Netherlands, the periods were chosen in such a way that they reflected: i) the period before the deposition level of 25 kg of nitrogen per hectare per year was exceeded (1956–1970), ii) the period in which the deposition levels peeked (1976–1990) and iii) the more recent period (1996–2010) during which the deposition levels were considerably (on average 27%, 20–39%) lower compared to the second period.

### Estimation of Species Richness Change and the Role of Spatial Scale

As shown by previous studies [22], [19], [20], evaluating richness change at different spatial scales may render very different results. A multi-scale approach allows to distinguish between changes due to large range expansions or contractions (which will affect richness values of multiple fine scale cells, and hence have a substantial effect on the mean change value at finer spatial scales, but no effect at country level), or to country-level extinctions of spatially restricted species and species introductions (which will affect coarse scale richness, while only influencing a few fine scale cells). Therefore, we repeated the analyses for variously sized spatial grids (1×1 km, 10×10 km, 20×20 km, 40×40 km, 80×80 km grid cells as well as for the whole country). To deal with the non-standardized nature of this data we followed the methods described in [20] that are based on the probability of species being detected under a given sampling effort. We obtained estimates of relative richness change per grid cell as well as an estimation of the associated error. Techniques from meta-analysis which allowed weighing each cell based on its uncertainty, i.e the inverse of variance [23], were then applied to obtain an overall weighted value of richness (*Qw*) and assess if such value was significantly different from zero [20]. These calculations were done using rma.uni function of the R package metaphor [24]. To check if the method completely corrected for bias due to differences in sampling efforts, we included the log of the relative difference of number of records as a covariate. To obtain unbiased estimates of richness change for each grid cell, we calculated the partial residuals after removing the effect of sampling effort for each cell (see [20]).

For these analyses we used only grid cells that followed several selection criteria that minimize the sensitivity of the results to the removal/addition of one grid cell: a) at least 30 records per period, b) a records-to-species ratio of at least 1.5 in each of the two time periods and c) for locations with low sampling effort in one of the periods (i.e. less than five times the maximum number of species in the country), a less than 10-fold difference in numbers of records between periods was required. Also, if a grid cell has a high number of records but only records of one single species in a given period, sampling in this grid-cell was likely targeted to that species, and therefore such grid cells was also excluded from the further analysis. This produced a total of grid cells as indicated in table 1. Also 10 year periods were examined to explore the possible trade off between the criteria used (supporting information, table S1).

**Table 1:** Orthoptera and plant data: Number of grid cells resulting from the criteria (see text of methods section) applied.

			Grid scale (Total nr. of cells)										
	Pre-period/Post-period (#records)		1 km (46700)		10 km (467)		20 km (108)		40 km (25)		80 km (6)		
**Orthoptera**	**1956–1970/1976–1990** (3389/18562)		1		17		22		15		6		
		**1976–1990/1996–2010** (18562/173747)		38		113		52		23		6	
**Plants**	**1956–1970/1976–1990** (194032/3508705)		287		104		39		14		6		
	**1976–1990/1996–2010** (3508705/2064061)		2840		348		99		27		6		

### Relations between *Orthoptera*, Plants and Nitrogen Deposition

**Figure 1:**
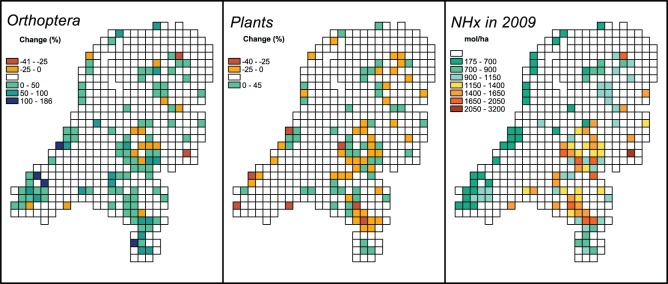
Changes in Orthoptera- and plant species richness between P2 and P3. **Ammonia deposition in 2009.** Plant species richness and ammonia deposition are only shown for those 10 by 10 km grid cells that met the criteria for Orthoptera data availability (see table 1).

For most of the ten by ten kilometre cells that met the criteria with respect to Orthoptera data availability (see table 1), the plant data were also sufficient to produce species richness change assessments using the same methods as used for Orthoptera (97 out of the 113 cells, see fig. 1). Nitrogen deposition (sum of reduced and oxidized nitrogen for the year 2009) was obtained for each grid cell in the Netherlands, based on a model developed by the Netherlands Environmental Assessment Agency (RIVM).

NHx deposition was more or less evenly distributed over the Netherlands during the first time period, showed a dramatic increase in certain areas in the second period (in relation to concentrations of livestock numbers, Noordijk & van der Hoek, unpublished data) after which it decreased again, but with an unchanged spatial distribution. Thus the 2009 data (fig. 1) also reflect the spatial pattern of the increase of NHx deposition between the first and second period and therefore are also relevant for the first versus second period species richness comparisons. In other words: the 2009 levels per cell reflect the increase between P1 and P2 and the actual levels between P2 and P3.

The effects of changes in plant species richness and of NHx deposition upon the changes in Orthoptera species richness were tested using a linear model (GLM), using plant richness change and nitrogen deposition as explanatory variables. Prior to that the data were log transformed and checked for normality and homogeneity of residuals variance. Spatial autocorrelation of Orthoptera species richness change, plant species change and nitrogen deposition was tested by comparing the fit of a null model with and without spatial autocorrelation structure. If residuals were significantly spatially auto-correlated, models were corrected accordingly (see [25]–[26]). Spatial autocorrelation was equally considered in all statistical analyses described hereafter.

Data processing and richness analyses were done in R [27].

### Role of Species Traits on Orthoptera Diversity Changes

A total of 47 species of Orthoptera were included in the analyses. To evaluate if diet breadth and type regulated Orthoptera community changes, these 47 species were divided into different groups according to: food type (herbivore in all life stages or not so) and food specificity (specialist herbivore or generalists). The food type and food specificity groups are nested (supporting information, table S2). The generalists group consists of six herbivorous species and 19 omnivorous species. The group of specialists consists of 21 herbivorous species and one omnivorous species (*Metrioptera roeselii*).

For the 2^nd^ and 3^rd^ time period comparison, both the trend analyses and the evaluation of the effects of plant species richness change and of ammonia deposition on Orthoptera richness change were also done for the trait-based subsets of Orthoptera species.

## Results

### Temporal Changes in Plant and *Orthoptera* Species Richness in the Netherlands

**Figure 2:**
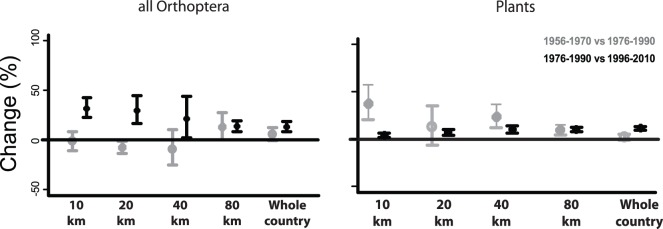
Trends in species richness change (ΔS% +−95% confidence intervals) for Orthoptera and plant species. Trends at the 10, 20, 40, 80 km grids and all Netherlands. In grey: changes between 1956–1970 and 1976–1990. In black: changes between 1976–1990 and 1996–2010. See table 1 for the numbers of grid cells. Filled symbols indicate change of significantly diffrent from zero; open symbols indicate that change was not significantly different from zero. A pair of results is considered not to be significantly different if the 95% error bars do not overlap.

Between 1956–1970 (P1) and 1976–1990 (P2) pronounced plant richness increases were detected at finer scales and no changes were detected at country level (fig. 2).

Between P2 and 1996–2010 (P3) changes were much less accentuated and only mild increases in richness were detected at all scales. When looking in more detail to the fine scale (10 km) changes in richness (fig. 1), our results show that increases in richness found between P2 and P3 were negatively related with ammonia (i.e. increases were stronger in regions with lower ammonia deposition; table 2a).

**Table 2:** Effect of Ammonia andPlants on Orthoptera species.

a) all species											
	Factor										
	NHx					Plants		NHx*Plants			
Response variable	P-value					P-value		P-value	df	AICc	ΔAICc
**Plants 1956–1970/1976–1990**											
Best model	−					n/a		n/a	104	76.0	0
Second best model (full model)	n.s. (P = 0.33)					n/a		n/a	103	77.1	1.1
**Best model**	**−**										
**Plants 1976–1990/1996–2010**											
Best model (full model)	**<0.03**				n/a			n/a	344	**−**47.8	0
Second best model	–				n/a			n/a	345	**−**45.2	2.6
**Best model**	Y = −0.00006× Nhx										
**Orthoptera 1956–1970/1976–1990**											
Best model	–			n.s. (P = 0.19)				–	6	4.9	0
Second best model	**0.0440**			**0.0166**				–	5	6.4	1.5
**Best model**	Y = 0.31159×Plants										
**Orthoptera 1976–1990/1996–2010**											
Best model (full model)	n.s. (**P = 0.064**)		–					–	95	−6.6	0
Second best model	–		–					–	96	−5.2	1.4
**Best model**	Y = −0.00009×Nhx										
**b) Orthopteraby subgroup** (**1976–1990/1996–2010**)											
*By food type subgroup:*											
**Herbivores**											
Best model (full model)	–	–						–	81	13.7	0
Second best model	–	n.s. (P = 0.20)						–	80	14.2	0.5
**Best model**	–										
**Non-herbivores**											
Best model	–	–						–	25	20.3	0
Second best model	–	n.s. (P = 0.51)						–	24	22.0	1.7
**Best model**	–										
*By food specificity subgroup:*											
**Specialists**											
Best model	–	–						–	82	2.9	0
Second best model	n.s. (P = 0.63)	–						–	81	4.8	1
**Best model**	–										
**Generalists**											
Best model (full model)	–	–					–		27	11.8	0
Second best model	n.s. (P = 0.35)	–					–		26	13.3	0
**Best model**	–										
**Habitat narrow**											
Best model (full model)	–	–					–		54	6.8	0
Second best model	–	ns (0.28)					–		53	7.8	1.0
**Best model**	–										
**Habitat wide**											
Best model (full model)	**0.0075**	–					–		59	−0.5	0
Second best model	**0.0105**	ns (0.59)					ns (**0.07**)		57	0.6	1.1
**Best model**	Y = −0.00014×***NHx***										
*By development rate subgroup:*											
**One year**											
Best model (full model)	–	–						–	87	13.3	0
Second best model	ns (0.61)	–						–	86	15.1	2.0
**Best model**	–										
**Two years**											
Best model (full model)	–	**0.0282**						–	9	−9.3	0
Second best model	ns (0.99)	**0.0286**						–	8	−7.5	1.8
**Best model**	Y = 0.34166×***Plants***										
*By dispersability subgroup:*											
**Dispersal limited**											
Best model (full model)	**0.0129**	–						–	41	17.7	0
Second best model	**0.0127**	ns (0.58)						–	40	19.7	2.0
**Best model**	Y = −0.00018×***NHx***										
**Dispersal high**											
Best model	–	–						–	63	−5.2	0
Second best model	ns (0.68)	–						–	62	−3.2	2
**Best model**	–										
*By Egg deposition subgroup:*											
**Eggs in soil**											
Best model	**0.0307**	–					–		93	−18.5	0
Second best model	**0.0256**	ns (0.34)					–		92	−17.3	1.2
**Best model**	Y = −0.00009×***NHx***										
**Eggs in plants**											
Best model	–	ns (0.12)					–		6	9.6	0
Second best model	ns(**0.06**)	**0.0354**					–		5	11.7	2.1
**Best model**	Y = 0.745467×***Plants***										

All variables analysed at the level of 10 km grid cells. Response variables (Plant richness change and Orthoptera richness change) were log transformed to normalize residuals: Orthoptera = log(orthoptera richness in post period/orthoptera richness in pre period); Plants = log (plant richness in post period/plant richness in pre period); Explanatory variables were: NHx (Ammonia deposition), Plants, Interaction NHx*Plants.The two most parsimonious models are listed according to AICc values and the equation of the best model (lowest AICc) is provided for each subset. P-values were obtained from a likelihood ratio test in which deviances with and without that term in the model were compared. n.s., P>0.05. Orthoptera species richness models and Nitrogen deposition models were corrected for spatial autocorrelation of residuals (exponential and spherical correlation structure, respectively). The residuals of plants species richness change models were not significantly spatially correlated. The symbol ‘–’ represents a variable not included in the model. n/a = not applicable.

**Figure 3:**
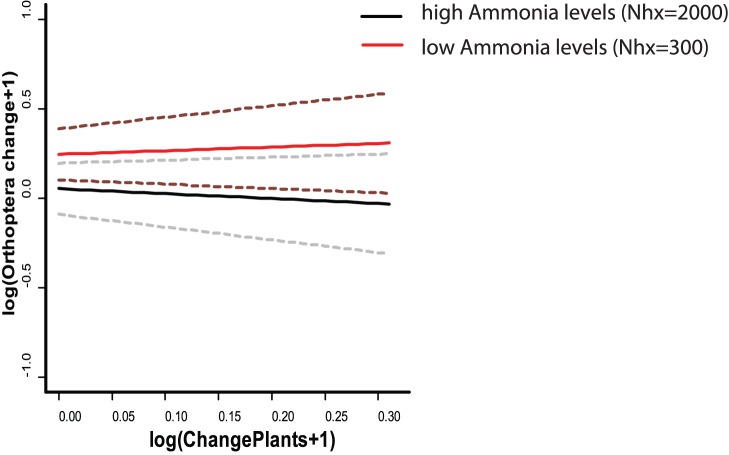
Orthoptera species richness change (+−SD, dashed lines) against plant species change at low versus high nitrogen levels (all Orthoptera). All comparisons between the second (1976–1990) and third time period (1996–2010) and in 10 km grid cells. The black lines show the estimated values for Orthoptera under a fixed value of ammonia; the grey lines represent the 95% confidence intervals. Ammonia levels in mol/ha.

Changes in Orthoptera species richness (S) between P1 and P2, were not accentuated i.e. small significant declines being only detected at 20 km scale, while at coarser scales increases were detected (fig. 2). Such increases were positively related with changes in plant richness (table 2a). Between P2 and P3 Orthoptera richness change (%) was significantly positive at all scales, increases being more pronounced at finer scales. Moreover, ammonia levels tended (P = 0.064) to be negatively related with Orthoptera richness changes detected between P2 and P3 (table 2a), but no significant effect of plant richness change on Orthoptera was detected. Therefore, increases of Orthoptera and plants richness were more frequent at low levels of ammonia, while at high levels of ammonia Orthoptera richness increases were much less accentuated (see fig. 3).

### Role of Species Traits on *Orthoptera* Diversity Changes

**Figure 4:**
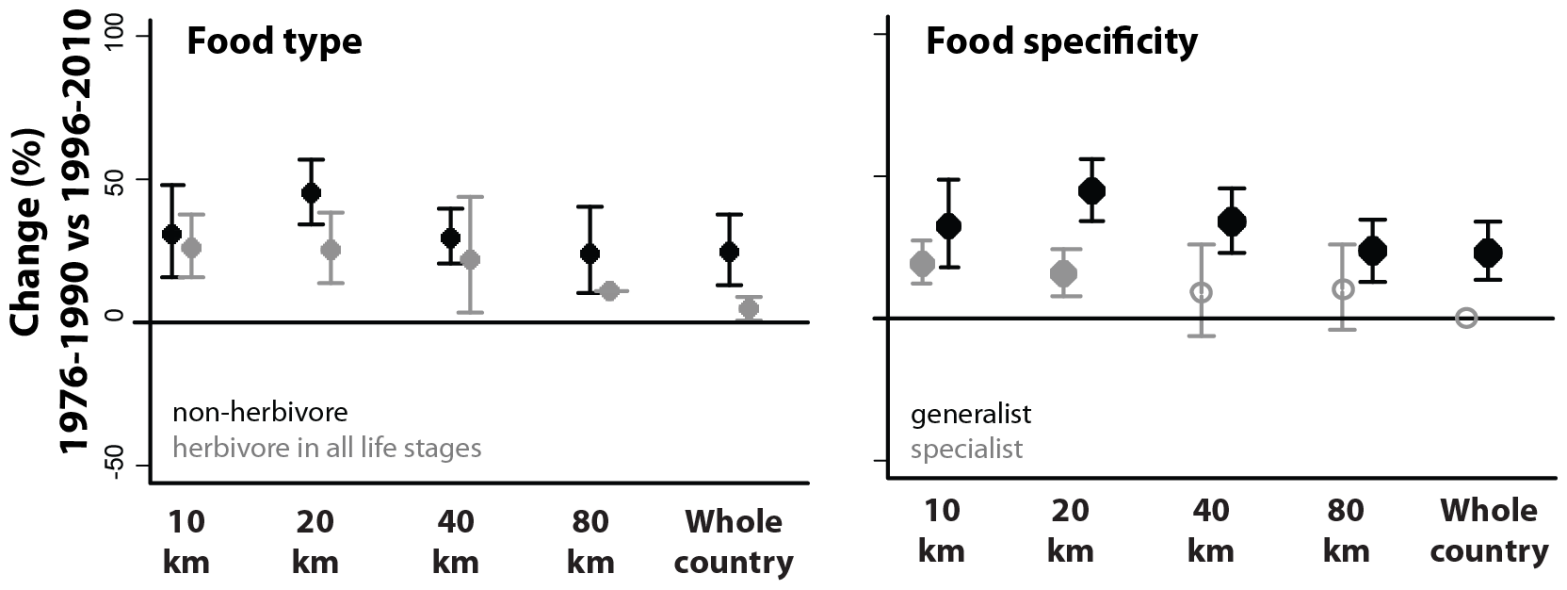
Trends in species richness change (ΔS% +−95% confidence intervals) for the food trait-based subsets of Orthoptera species. Trends at the 10, 20, 40, 80 km grids and whole country. See table S3 of the supporting material for the numbers of grid cells. Filled symbols indicate change of significantly diffrent from zero; open symbols indicate that change was not significantly different from zero. A pair of results is considered not to be significantly different if the error bars do not overlap.

**Figure 5:**
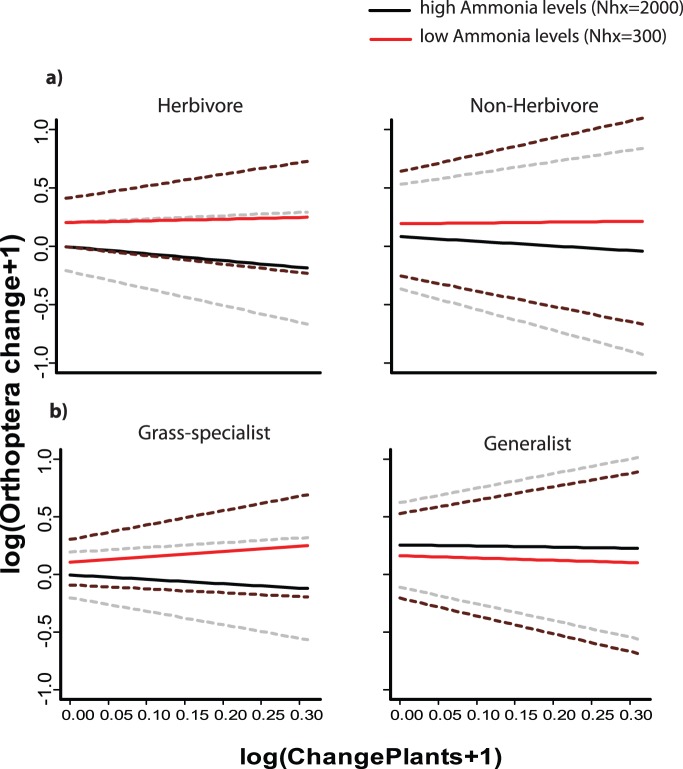
Orthoptera species richness change (+−SD, dashed lines) against plant species change at low versus high nitrogen levels (food trait subgroups). All comparisons between the second (1976–1990) and third time period (1996–2010) and in 10 km grid cells. The black lines show the estimated values for Orthoptera under a fixed value of ammonia; the grey lines represent the 95% confidence intervals. Ammonia levels in mol/ha.

Between P2 (1976–1990) and P3 (1996–2010) herbivores and non-herbivore Orthoptera species had similar patterns of change, the last group only having slightly more pronounced richness increases particularly at coarser scales (see fig. 4). Food specialization had, however, a stronger influence on the patterns of richness change found between P2 and P3, increases being more accentuated for generalists than for grass specialists. No significant changes in richness were found for specialist at coarser scales (40 km to country level), while generalist richness increased significantly at all spatial scales. When looking in more detail at finer scale, we found no significant effect of plant richness change on none of the food type trait groups of Orthoptera (table 2b), but for herbivores and grass specialists richness was likely to increase under low ammonia levels, grass specialists also being more responsive to changes in plant richness (see fig. 5).

**Figure 6:**
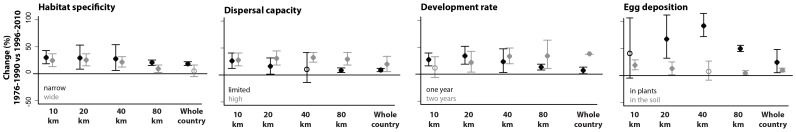
Trends in species richness change (ΔS% +−95% confidence interval) for the non-food trait based subsets of Orthoptera species. Trends at the 10, 20, 40, 80 km grids and whole country. See table S3 of the supporting material for the numbers of grid cells. Filled symbols indicate change of significantly diffrent from zero; open symbols indicate that change was not significantly different from zero. A pair of results is considered not to be significantly different if the error bars do not overlap.

As for non-food traits analyses, our results show that at course-scale levels (80 km and the country level) species with a narrow habitat range had more accentuated increases than species with wide habitat range. At finer scales, both wide and narrow habitat range groups increased in richness (fig. 6) and when looking in more detail at fine scale changes, ammonia levels only had a negative effect on richness change of Orthoptera species with a wide (rather than narrow) habitat range (table 2b).

Dispersal ability also seems to influence patterns of richness change. At a coarse scale (80 km) increases in species richness were significantly more accentuated for species with higher dispersal ability (fig. 6). At finer scales, both groups increased in richness (fig. 6), but for Orthoptera species with limited (rather than high) dispersal ability richness change was negatively affected by ammonia levels (table 2b).

Species with a development rate of two years had a higher increase in species richness at the country level than species with a one year development rate. At finer scales increases in richness were only detected for the group with one year development, changes of assemblages with a two year development rate being dependent on plant species richness increases (table 2b). The positive effect of plants was not detected for the subgroup of species with a one year development.

Orthoptera species that deposit their eggs in plants had substantially more pronounced increases in richness between P2 and P3 than species that oviposit in bare soil (at 20, 40 and 80 km levels). At finer scales, increases for species that lay eggs on bare soil were constrained by high ammonia while richness change of the group of species that deposit their eggs in plants was positively related with changes in plant assemblages (table 2b).

## Discussion

Nitrogen availability has increased substantially over the past century. While many studies have focused on its impact on plant communities, little is known about the effects on higher trophic levels. Here we show that both plants and herbivores are negatively affected by nitrogen deposition; this effect being particularly marked for species that have a broad habitat range and limited dispersal ability, as well as on species that deposit their eggs in the soil.

### Changes in Species Richness during Time Periods of High and Lower Nitrogen Levels

We expected plant species richness to be negatively affected by high levels of nitrogen. While the results for P1 (1956–1970) versus P2 (1976–1990) were not conclusive, this expectation was confirmed by the changes between P2 and P3 (1996–2010).

Although fine scale changes in plant richness were more accentuated during the time period where NHx (ammonia) deposition was increasing, i.e. between P1 and P2, spatially explicit analyses showed that between P1 and P2 patterns of plant richness change are not significantly related to NHx values in 2009 (Table 2a), suggesting that although both plant richness and NHx increased between P1 and P2, no causal relationship exists. It is, however, possible that our assumption regarding the 2009 nitrogen deposition data to also reflect the spatial pattern of the increase of NHx deposition between the first and second period does not fully hold. Furthermore it is likely that plant species with different physiological requirements react differently to ammonia levels. More detailed analyses that take into consideration nitrophily of plants would be required to fully explore the effect of nitrogen on plants. Also the statistical power for the spatial analysis of plant and nitrogen changes between P1 and P2 (n = 7) is considerably lower than for P2 versus P3 (n = 97).

As mentioned above, the results for P2 versus P3 suggest that the overall effect of nitrogen is negative, since they show that plant richness increases are most accentuated in cells with low NHx levels. This negative effect is constraining the overall species richness increases between P2 and P3 which may be driven by other drivers (e.g. landuse changes, climate, ecological restoration efforts).

As for Orthoptera, we expected overall changes in species richness to parallel changes in nitrogen deposition and changes in plant species richness both in time and space. This was confirmed by the results of P1 versus P2, although the statistical power in that part of the analysis was low (table 2a). Indeed, between P2 and P3 levels of NHx tended to be negatively associated with Orthoptera richness change (Fig. 3, table 2a). However, no spatially explicit relation between changes in plant species richness and changes in Orthoptera species richness was found (table 2a). Therefore, while the negative impact of nitrogen propagates to higher trophic levels, the general impact via plants as formulated in the first hypothesis is not clear. This could be due to the fact that not all Orthoptera species are herbivores, or because other life history traits also play a role. We discuss the effect of such traits below. Moreover, changes in abundance of species may vary substantially with no change in species richness, many species persisting in a given location with very few individuals. It is therefore possible, that an effect of plants would only be detected with detailed information on the changes of plant biomass.

### Aspects of Scale

Changes in Orthoptera species diversity in the Netherlands during the study period in addition to nitrogen are also driven by two other main factors: habitat changes [28] and climate changes [29]–[31]. The increase of Orthoptera species richness between between P2 (1976–1990) and P3 (1996–2010) at the fine scales among our results, is most probably related to factors that lead to large changes in species range, while the increase at country level reflects the incidental arrival of newcomers. Indeed, the significant increases at coarser scales (country level and 80 km) between P1 (1956–1970) and P2 reflect the appearance of a new species in the Netherlands (e.g. *Conocephalus discolor*, [32]). Between P2 and P3, increases at country level were still detected, suggesting that further species arrived (e.g. *Sphingonotus caerulans*, [33]).

An overall positive trend of species richness at a certain scale due to new arrivals, does not rule out the possibility that at the same time (local) abundances of individual species at the same or lower scale levels have gone down. Indeed, while we detected increases in richness between P1 and P2 several species are known to have reduced their local abundances [8]. Also increases in finer scale richness due to expansion of a few species might buffer declines in richness due to local extinctions of other species. Assessing whether species assemblages are becoming more homogenized in space would shed light on whether this indeed is occurring.

### Influence of Food Type and Food Specificity Traits

Species responses may depend on their traits. Therefore, we explored the possible mechanisms by comparing responses of groups with contrasting traits.

Contrary to our expectations (hypothesis 2), herbivores were not significantly more impacted than non-herbivores. However, we did find a negative trend (although non significant) between species richness change among herbivorous Orthoptera and plant species richness change at higher levels of NHx deposition, whereas such relation could not be detected for the group of non-herbivores (carni- and omnivores; fig. 5a). The fact that the diversity of herbivorous Orthoptera under high levels of NHx deposition is going down in 10 km grid cells where plant diversity is increasing, is an indication that indeed also food quality changes in relation to changing internal C:N ratios of plants due to elevated nitrogen depositions might play a role.

Grass specialists compared to generalists do show a lower species diversity change at some of the levels of scale between P2 and P3 (during which the nitrogen overload was decreasing). This result is in line with hypothesis 3 that specialists compared to generalists would be less impacted by changes in nitrogen deposition. However, we also found a (non significant) negative trend between species richness change among specialists and plant species richness change at higher levels of NHx deposition, whereas such relation could not be detected for the group of generalists. This indicates that it is the group of specialist that is more impacted by higher NHx deposition levels. Plant species diversity increased between P2 and P3, even so in those grid cells with the highest deposition levels (fig. 5b, NHx = 2000), whereas under high deposition conditions the (grass specialist) herbivore Orthoptera seem not to profit from improving environmental conditions between P2 and P3, which is in contrast to hypothesis 3. The fuzzy results for the food specialisation traits may be related to the actual nature of the groups. ‘Specialist’ in this study is synonym to ‘specialist herbivore’. In the case of the Dutch Orthoptera species this does not mean that we are talking about highly specific co-evolved plant-host relationships, but rather a preference of these species for grasses and sedges (or in two species: mosses and algae). Furthermore the results with respect to the food traits are potentially phylogenetically constrained. Both the group of food specialists and group of herbivores are namely overrepresented by species from two genera (*Chorthippus* and *Tetrix*). Therefore, we cannot exclude the possibility that another, not yet analyzed, trait that the members of this group share might be responsible for the observed trends.

### Influence of Non-food Related Traits

While food related traits do not clearly explain the species diversity changes, non-food related traits significantly explained part of the variation for Orthoptera community trends.

The significantly higher species diversity increase among species with narrow habitat preferences, high dispersal capacity or two year development rate at the 80 km and/or whole country scale, is likely related with the arrival of a new species (e.g. *S. caerulans*) in the study area.

Furthermore, the spatially explicit analyses at the 10 km scale level showed a significantly negative effect of NHx among Orthoptera species with wide habitat range (table 2b), which is not present among the narrow habitat species. This may be interpreted as habitat quality (in this case nitrogen deposition) being of secondary importance to the overall availability of suitable habitats (for habitat specialists). The fact that the significant negative effect of NHx is only found within the group of species with low dispersal ability and not among Orthoptera with high dispersal capacity (table 2b) may be explained by the higher ability of the latter species to escape non-favourable local conditions. The fourth hypothesis, a wide habitat range and limited dispersal ability to show a stronger relationship with changes in nitrogen deposition than habitat specialists, thus was confirmed.

The positive relationship between the species richness change among Orthoptera with a two year development rate and plant species richness change may be an indication of the higher dependency of these Orthoptera species on the availability of good quality habitat in order for the nymphs to survive an extra year of development before being able to migrate as adults.

The results on egg deposition (in plants versus in the soil) provide further clues to unravel the effects of nitrogen deposition upon Orthoptera species richness change. The higher increase of species richness between P2 and P3 among Orthoptera species that deposit their eggs in plants (at different scale levels) and the significantly negative effect of NHx on the subgroup of Orthoptera species that deposit their eggs in the soil (table 2b), both point in the direction of a vegetation structure link between nitrogen deposition and Orthoptera species richness. Hypothesis 5 (that high nitrogen deposition leads to a more dense vegetation that is also be less suitable to soil laying Orthoptera species because of the less favourable microclimate), is thus confirmed.

### Concluding Remarks

Notwithstanding the necessity of further steps and refinements, we can conclude that the application of species richness change analysis to this group of species provides an interesting additional tool for answering ecological questions regarding the impact of nutrient availability on plant herbivore interactions. Ammonia deposition indeed does not only affect plant communities but also propagates to higher trophic levels.

The negative effect of nitrogen on Orthoptera could even be demonstrated during times of decreasing nitrogen levels (after 1990). This suggests that nitrogen deposition levels in the Netherlands in the period 1996–2010 were still exceeding the critical deposition levels [3](Bobbink et al. 2011) for many natural and semi-natural ecosystems. However it is also possible that there is a time-lag in the response of Orthoptera community, the negative effects of past time periods where nitrogen deposition was higher only translating into changes in species richness in the period 1996–2010.

The observed patterns of species richness change could, to a large extent, be explained by traits of the Orthoptera species. The lack of significant effect of plant richness on Orthoptera change does not overrule the possibility that this group of herbivores is affected by subtle changes in plant abundance and plant quality. But overall habitat specificity, dispersal capacity and the location of egg deposition had a stronger influence than food related traits, negative effects of nitrogen being only evident in certain trait groups. Life history traits are therefore essential to fully evaluate the negative effects of nitrogen deposition.

## Supporting Information

Table S1 **Number of grid cells resulting from applying the criteria in case of ten-year periods.** As an alternative to the fifteen-year periods, also ten-year periods were examined to explore the possible trade off between the criteria (see text of methods section) used. Limiting the fifteen-year periods by removing the five earliest years decreases the chance of meeting criterion a) but increases the chance of meeting criterion c). The ten year-periods produced a lower number of grid cells compared to the fifteen year periods.
(DOC)

Table S2 **Orthoptera traits.** Data on Orthoptera species traits, derived from the distribution atlas [8] and from the expert knowledge of European Invertebrate Survey - Netherlands.
(DOC)

Table S3 **Number of grid cells resulting from applying the criteria to the trait-based subsets of Orthoptera species.** See text of methods section for a description of the criteria (a-c). The trait-based subsets of Orthoptera species logically produced lower numbers of grid cells compared to the full set of Orthoptera species (Table 1).
(DOC)
