# Supplementary material for: Temporal-Spatial Dynamics in Orthoptera in Relation to Nutrient Availability and Plant Species Richness
Source: PLoS One. 2013 Aug 12;8(8):e71736. doi: 10.1371/journal.pone.0071736 (PMC3741129; doi:10.1371/journal.pone.0071736)
Supplement: Table S1 — Number of grid cells resulting from applying the criteria in case of ten-year periods. As an alternative to the fifteen-year periods, also ten-year periods were examined to explore the possible trade off between the criteria (see text of methods section) used. Limiting the fifteen-year periods by removing the five earliest years decreases the chance of meeting criterion a) but increases the chance of meeting criterion c). The ten year-periods produced a lower number of grid cells compared to the fifteen year periods. (DOC) [file pone.0071736.s001.doc]

**Table S1.** Number of grid cells resulting from the criteria applied in case of ten-year periods

| **Pre-period/Post-period (#records)** | Grid scale(Total nr. of cells) | *1 * 1 (46700)* | *10*10 (467)* | *20*20 (108)* | *40*40 (25)* | *80*80 (6)* |
| --- | --- | --- | --- | --- | --- | --- |
| Orthoptera Ten year periods | |  |  |  |  |  |
| 1961-1970/1981-1990 (2833/17568) | | - | 12 | 17 | 13 | 5 |
| 1981-1990/2001-2010 (17568/151616) | | 32 | 104 | 51 | 23 | 6 |
|  | |  |  |  |  |  |
