# Supplementary material for: Temporal-Spatial Dynamics in Orthoptera in Relation to Nutrient Availability and Plant Species Richness
Source: PLoS One. 2013 Aug 12;8(8):e71736. doi: 10.1371/journal.pone.0071736 (PMC3741129; doi:10.1371/journal.pone.0071736)
Supplement: Table S2 — Orthoptera traits. Data on Orthoptera species traits, derived from the distribution atlas [8] and from the expert knowledge of European Invertebrate Survey - Netherlands. (DOC) [file pone.0071736.s002.doc]

**Table S2. Orthoptera traits.**

| *species* | *Development rate (year)* | *Habitat specifity* | *Dispersal capacity* | *Herbivore in all life stages* | *Food specifity* | *Egg deposition* |
| --- | --- | --- | --- | --- | --- | --- |
| *Acheta domesticus* | One | narrow | limited | no | generalist | soil |
| *Chorthippus albomarginatus* | One | narrow | limited | yes | specialist | soil |
| *Chorthippus apricarius* | One | narrow | limited | yes | specialist | soil |
| *Chorthippus biguttulus* | One | wide | high | yes | specialist | soil |
| *Chorthippus brunneus* | One | wide | high | yes | specialist | soil |
| *Chorthippus dorsatus* | One | narrow | limited | yes | specialist | soil |
| *Chorthippus mollis* | One | wide | limited | yes | specialist | soil |
| *Chorthippus montanus* | One | wide | limited | yes | specialist | soil |
| *Chorthippus parallelus* | One | narrow | high | yes | specialist | soil |
| *Chorthippus vagans* | One | wide | limited | yes | specialist | soil |
| *Chrysochraon dispar* | One | narrow | limited | yes | specialist | plants |
| *Conocephalus discolor* | One | wide | high | no | generalist | plants |
| *Conocephalus dorsalis* | One | wide | high | no | generalist | plants |
| *Decticus verrucivorus* | Two | wide | limited | no | generalist | soil |
| *Ephippiger ephippiger* | Two | narrow | limited | no | generalist | soil |
| *Gampsocleis glabra* | Two | narrow | limited | no | generalist | soil |
| *Gomphocerippus rufus* | One | narrow | high | yes | specialist | soil |
| *Gryllotalpa gryllotalpa* | Two | narrow | limited | no | generalist | soil |
| *Gryllus campestris* | One | arrow | high | no | generalist | soil |
| *Leptophyes punctatissima* | One | wide | limited | yes | generalist | plants |
| *Locusta migratoria* | One | wide | high | yes | generalist | soil |
| *Meconema meridionale* | Two | narrow | limited | no | generalist | plants |
| *Meconema thalassinum* | One | wide | high | no | generalist | plants |
| *Metrioptera bicolor* | Two | narrow | high | no | generalist | plants |
| *Metrioptera brachyptera* | Two | narrow | limited | no | generalist | soil |
| *Metrioptera roeselii* | One | narrow | high | no | specialist | plants |
| *Myrmeleotettix maculatus* | One | narrow | limited | yes | specialist | soil |
| *Nemobius sylvestris* | Two | narrow | limited | no | generalist | soil |
| *Oedipoda caerulescens* | One | narrow | limited | yes | generalist | soil |
| *Omocestus rufipes* | One | narrow | limited | yes | specialist | soil |
| *Omocestus viridulus* | One | narrow | limited | yes | specialist | soil |
| *Phaneroptera falcata* | One | narrow | high | yes | generalist | soil |
| *Pholidoptera griseoaptera* | Two | wide | limited | no | generalist | soil |
| *Platycleis albopunctata* | One | narrow | high | no | generalist | soil |
| *Psophus stridulus* | One | narrow | limited | yes | generalist | soil |
| *Sphingonotus caerulans* | One | narrow | high | no | generalist | soil |
| *Stenobothrus lineatus* | One | narrow | limited | yes | specialist | soil |
| *Stenobothrus stigmaticus* | One | narrow | limited | yes | specialist | soil |
| *Stethophyma grossum* | One | wide | limited | yes | specialist | soil |
| *Tachycinesa synamorus* | One | narrow | limited | no | generalist | soil |
| *Tetrix bipunctata* | One | narrow | limited | yes | specialist | soil |
| *Tetrix ceperoi* | One | narrow | high | yes | specialist | soil |
| *Tetrix subulata* | One | wide | high | yes | specialist | soil |
| *Tetrix tenuicornis* | One | wide | limited | yes | generalist | soil |
| *Tetrix undulata* | One | wide | limited | yes | specialist | soil |
| *Tettigonia cantans* | Two | narrow | limited | no | generalist | soil |
| *Tettigonia viridissima* | Two | wide | high | no | generalist | soil |
