# Supplementary material for: Temporal-Spatial Dynamics in Orthoptera in Relation to Nutrient Availability and Plant Species Richness
Source: PLoS One. 2013 Aug 12;8(8):e71736. doi: 10.1371/journal.pone.0071736 (PMC3741129; doi:10.1371/journal.pone.0071736)
Supplement: Table S3 — Number of grid cells resulting from applying the criteria to the trait-based subsets of Orthoptera species. See text of methods section for a description of the criteria (a-c). The trait-based subsets of Orthoptera species logically produced lower numbers of grid cells compared to the full set of Orthoptera species (Table 1). (DOC) [file pone.0071736.s003.doc]

**Table S3. Number of grid cells resulting from the criteria (see text of methods section) applied**

| **Pre-period/Post-period (trait)** | Grid scale(Total nr. of cells) | *1 * 1 (46700)* | *10*10 (467)* | *20*20 (108)* | *40*40 (25)* | *80*80 (6)* |
| --- | --- | --- | --- | --- | --- | --- |
| **Food type** | |  |  |  |  |  |
| 1956-1970/1976-1990 (carnivore or omnivore) | | - | 3 | 7 | 7 | 4 |
| 1956-1970/1976-1990 (herbivore in all life stages) | | - | 11 | 15 | 12 | 6 |
| 1976-1990/1996-2010 (carnivore or omnivore) | | 2 | 30 | 35 | 16 | 6 |
| 1976-1990/1996-2010 (herbivore in all life stages) | | 21 | 85 | 52 | 24 | 6 |
| **Food specificity** | |  |  |  |  |  |
| 1956-1970/1976-1990 (generalist) | | - | 3 | 7 | 6 | 5 |
| 1956-1970/1976-1990 (specialist) | | - | 11 | 15 | 11 | 6 |
| 1976-1990/1996-2010 (generalist) | | 3 | 32 | 32 | 14 | 6 |
| 1976-1990/1996-2010 (specialist) | | 20 | 88 | 56 | 24 | 6 |
| **Habitat specificity** | |  |  |  |  |  |
| 1956-1970/1976-1990 (narrow) | | - | 7 | 11 | 9 | 5 |
| 1956-1970/1976-1990 (wide) | | - | 8 | 8 | 8 | 6 |
| 1976-1990/1996-2010 (narrow) | | 12 | 60 | 45 | 21 | 6 |
| 1976-1990/1996-2010 (wide) | | 10 | 65 | 46 | 25 | 6 |
| **Dispersal capacity** | |  |  |  |  |  |
| 1956-1970/1976-1990 (limited) | | - | 8 | 12 | 10 | 5 |
| 1956-1970/1976-1990 (high) | | - | 4 | 8 | 9 | 6 |
| 1976-1990/1996-2010 (limited) | | 11 | 43 | 42 | 21 | 6 |
| 1976-1990/1996-2010 (high) | | 11 | 72 | 48 | 25 | 6 |
| **Development rate** | |  |  |  |  |  |
| 1956-1970/1976-1990 (one year) | | - | 15 | 17 | 14 | 6 |
| 1956-1970/1976-1990 (two years) | | - | - | 1 | 5 | 2 |
| 1976-1990/1996-2010 (one year) | | 30 | 97 | 50 | 24 | 6 |
| 1976-1990/1996-2010 (two years) | | - | 11 | 17 | 10 | 5 |
| **Egg deposition** | |  |  |  |  |  |
| 1956-1970/1976-1990 (in soil) | | - | 13 | 18 | 15 | 6 |
| 1956-1970/1976-1990 (in plants) | | - | 1 | 1 | 3 | 4 |
| 1976-1990/1996-2010 (in soil) | | 26 | 106 | 55 | 23 | 6 |
| 1976-1990/1996-2010 (in plants) | | 1 | 9 | 13 | 14 | 6 |
|  | |  |  |  |  |  |
